# Image feature extraction and recognition model construction of coal and gangue based on image processing technology

**DOI:** 10.1038/s41598-022-25496-5

**Published:** 2022-12-05

**Authors:** Lei Zhang, YiPing Sui, HaoSheng Wang, ShangKai Hao, NingBo Zhang

**Affiliations:** 1 Key Laboratory of Deep Coal Mining of the Ministry of Education, School of Mines, China University of Mining and Technology, Xuzhou, 221116 China; 2 College of Coal Engineering, Shanxi Datong University, Datong, 037003 Shanxi China

**Keywords:** Mineralogy, Coal

## Abstract

Using image recognition technology to realize coal gangue recognition is one of the development directions of intelligent fully mechanized caving mining. Aiming at the problem of low accuracy of coal gangue recognition in fully mechanized caving mining, the extraction method of Coal and gangue images features is proposed, and the corresponding coal gangue recognition model is constructed. The illuminance value is an important factor affecting the imaging quality. Therefore, a multi-light source image acquisition system is designed, and the optimal illuminance value suitable for coal and gangue images acquisition is determined to be 17,130 Lux. There is a large amount of image noise in the gray-sc5ale image, so Gaussian filtering is used to eliminate the noise in the gray-scale image of coal and gangue. Then, six gray-scale features and four texture features are extracted from 900 coal and gangue images respectively. It is concluded that the three kinds of features of gray skewness, gray variance and texture contrast have the highest discrimination on coal and gangue images. Least squares vector machine has a strong ability to classify, so the use of least squares vector machine to achieve coal gangue identification, and build coal gangue identification model. The results show that the recognition accuracy of the model for coal gangue images is 92.2% and 91.5%, respectively, with gray skewness and texture contrast as indicators. This study provides a reliable theoretical support for solving the problem of low recognition rate of coal gangue in fully mechanized caving mining.

## Introduction

Comprehensive mechanized top coal caving mining technology is the key technology for mining thick and extra-thick coal seams in China. With the rapid development of artificial intelligence technology, how to complete the coal caving process with more intelligent and automatic technical means has become a hot topic of current research^1^. In particular, the development of image recognition technology provides new technical support for coal caving automation technology^2–4^. The coal caving automation technology is beneficial to realize the balance between coal mining and coal caving in coal mine underground working face. It reduces the labor intensity and casualty risk of miners and increases the production efficiency of coal mines^5,6^. The technology has high research value. At present, the automation technology of coal caving is restricted by the development of coal gangue identification technology^7^. Researchers have been looking for scientific means to realize coal gangue identification. Referring to the existing research results, the technical means to realize coal gangue identification mainly include thermal infrared technology, X-ray, natural γ-ray, ultrasonic and so on^8–10^. Nowadays, deep learning has made significant technological breakthroughs in the field of image recognition^11^. The speed and accuracy of computer image recognition have been significantly improved. Therefore, using image recognition technology to realize the separation of coal and gangue in coal production has certain practicability^12,13^. Professor Shan Pengfei adopted a coal-rock identification method based on machine deep learning FasterR-CNN, which realized the accurate identification and location of coal seam and rock stratum in coal-rock images^14^. Professor Wang Jiachen used the classical image processing algorithm to calculate the mixed gangue rate. This method has been widely used in the field of image recognition of coal gangue separation, and its reliability has been verified^15^. Professor Zhao Haodi studied coal gangue sorting based on machine vision technology, built a complete framework of coal gangue identification and positioning system based on machine vision, and finally realized coal gangue identification and precise positioning based on machine vision^16^. In conclusion, the use of machine vision for coal gangue recognition has good accuracy and high research value.

Compared with coal and gangue, color and texture are the most obvious differences in appearance. In most cases, coal is darker and more glossy than gangue ; in terms of texture, the texture of coal is more obvious, and there are more cracks on the surface, and the bright spots on the surface of coal during reflection can be effectively distinguished from gangue^17,18^. Therefore, it is scientific to study the differences in gray and texture features of coal and gangue images as indicators for distinguishing coal and gangue^19^. In the experiment, the coal and gangue were photographed, and the obtained images were grayed to construct the gray image database of coal and gangue. Then Gaussian filtering is performed on the gray image of coal and gangue to eliminate the noise in the digital image. The experiment extracts the characteristic value of coal and gangue image after image graying and Gaussian filtering^20,21^. The experimental results were plotted into a box-plot to study the numerical differences between coal and gangue images in various gray and texture features^22^. In the experiment, the gray and texture features with large difference are selected as the basis for coal and gangue image recognition, and the LS-SVM model is established^23^. At the same time, a large number of training is used to train it to improve the recognition accuracy of LS-SVM model. According to the above experimental research, a complete coal gangue identification system based on image processing technology can be constructed. The research adopts a novel perspective based on the grayscale features of coal and gangue images, that is, texture features, introduces the least squares support vector machine for coal gangue recognition, and studies the method of vector machine model to improve the correct rate of coal gangue recognition. It can be seen that the research is highly original and scientific.

## Difference analysis of coal and gangue image features

### Multi-light source image acquisition system for coal and gangue

In order to study the specific differences in gray and texture features between coal and gangue images, the database of coal and gangue images should be established first. After on-the-spot investigation, Xiaoyu Coal Mine in Shanxi Province was selected as the collection site of coal and gangue samples, and 300 pieces of coal and gangue were selected as experimental samples. These coal and gangue samples are mostly 15–20 cm in size, and the coal is mostly fat coal, gangue is mostly sandstone gangue. At the same time, through field investigation, it is found that Shanxi Xiaoyu Coal Mine is a coal mine with high gangue content in production, which also confirms the necessity of coal gangue identification technology.

Illumination is a key factor affecting image quality and related parameters. For example, when the same coal or gangue is taken under different lighting conditions, the image quality and related parameters will be different due to different illuminance. In order to use mature image recognition algorithm instead of manual to identify coal and gangue, before extracting relevant parameters of coal and gangue image, the illumination conditions that are most suitable for coal and gangue image acquisition experiment should be determined first.

**Figure 1:**
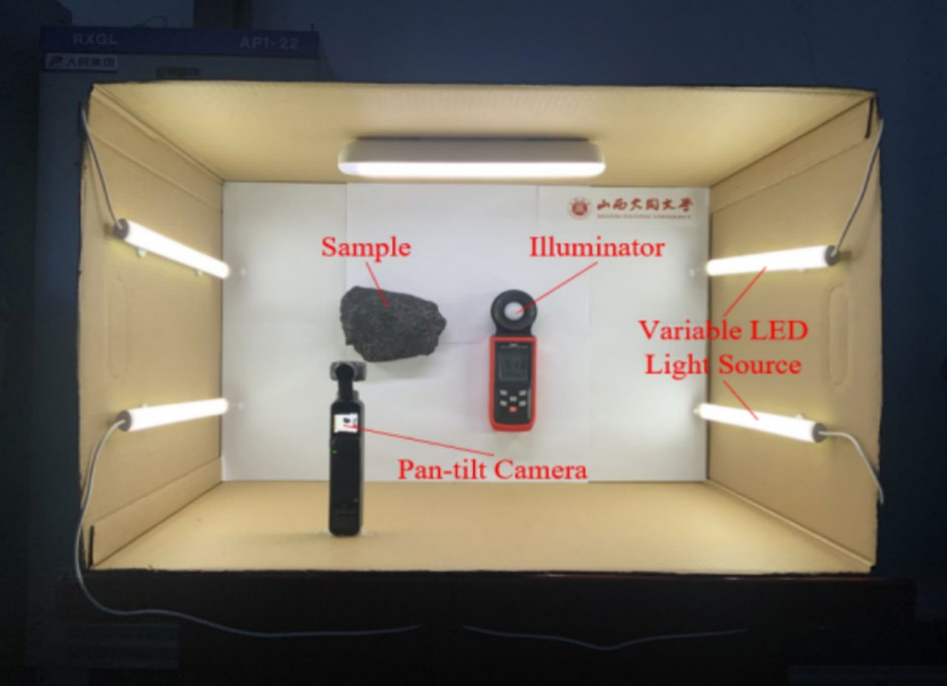
Multi-light source coal and gangue image acquisition system.

In order to obtain coal and gangue images under different illumination conditions required for the experiment, the following multi-source coal and gangue image acquisition system is designed. The image acquisition system uses three side LED variable light source, which can adjust the light intensity in the experiment in real time. In order to ensure that the value displayed in the illuminance meter is the accurate illuminance value of the coal and gangue sample surface, the coal and gangue sample is fixed at the height of the same level as the illuminance meter probe. The illuminance meter used in the experiment can detect the illuminance value of coal and gangue sample surface in real time, the maximum range is 100,000 Lux, and the resolution is 0.1 Lux. In the experiment, the three-side LED lights should be used as the only light source to control the light intensity of different gears. Therefore, the experiment should be carried out in the dark box, as shown in Fig. 1.

### Grayscale processing of coal and gangue images

In the processing of color images, the three components of R, G and B should be processed respectively, but in fact they cannot reflect the morphological characteristics of the image, and only the color is allocated from the optical principle. Many recognition algorithms are not strongly dependent on color. After image graying, with the decrease of matrix dimension, the corresponding calculation speed will be greatly improved, and the gradient information will be retained. According to the formula (1), the sub-image is grayed, and the operation speed is greatly improved while retaining the pixel gradient information. where R, G and B are the red, green and blue color values of pixels .

1 $$Gray = \left( {R^{*} 299 + G^{*} 587 + B^{*} 144 + 500} \right)/1000.$$

**Figure 2:**
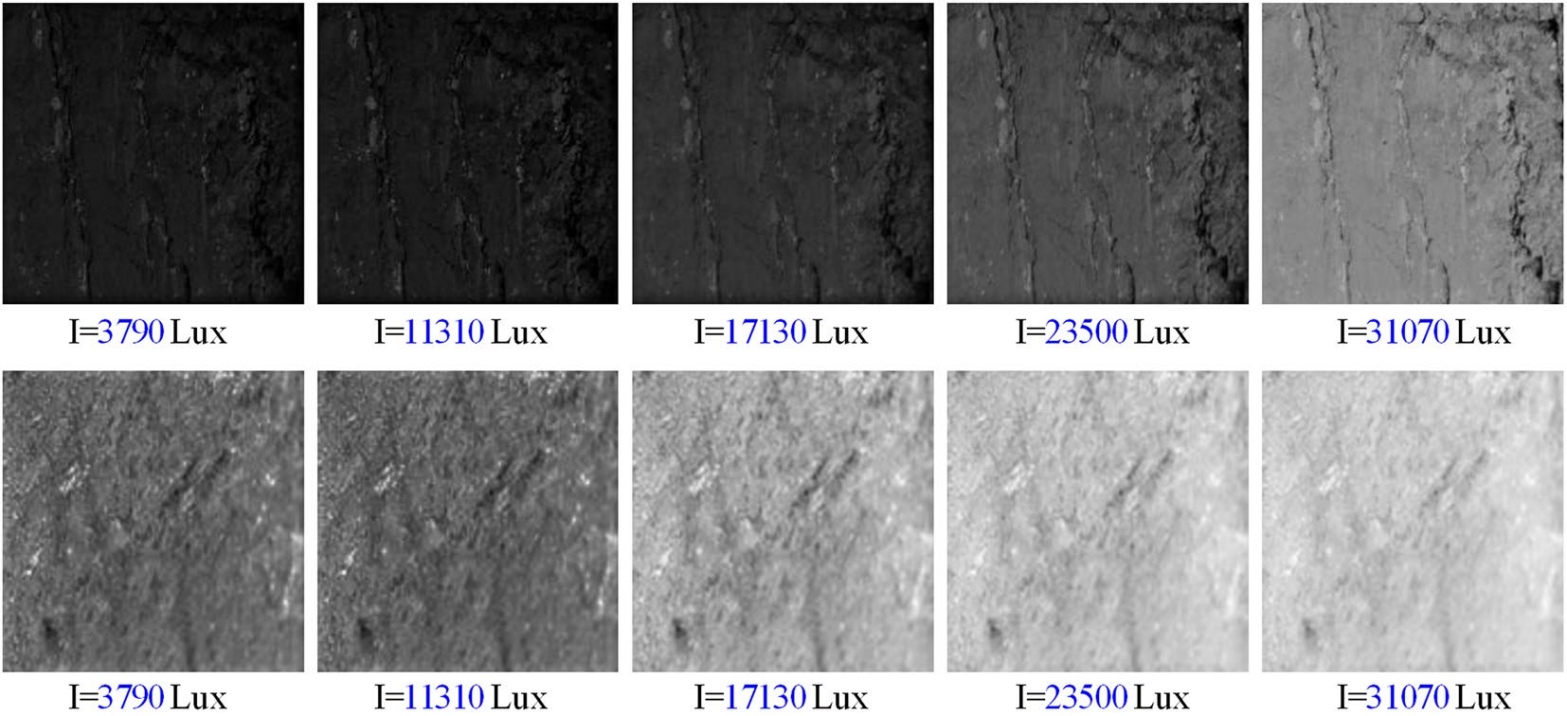
Grayscale sub-image of coal and gangue.

The five different illuminance involved in this experiment were 3790, 11,310, 17,130, 23,500 and 31,070 Lux, respectively. A total of 1800 coal and gangue images after graying were obtained in the experiment. Cut each image as a 640px × 480px subimage containing only samples without edges and backgrounds. The illumination conditions that are most suitable for the experiment should be determined before the gray and texture feature values of coal and gangue images are extracted. In the experiment, the images of coal and gangue with the most obvious color difference are selected for gray processing. The gray sub-images of coal and gangue obtained under different illumination conditions are shown in Fig. 2. The above five images are grayscale images of coal obtained under different illuminance values, and the following five images are grayscale images of gangue obtained under different morning readings.

A pixel in the gray image is represented by the gray level within the range of 0–255. The smaller the gray level is, the darker the image is. The larger the gray level is, the grayer the image is. By observing the gray-scale images of coal and gangue, it is found that under the same illumination condition, the color of coal is darker and the surface cracks are more obvious, while the gangue is gray–white and the surface is smoother than coal. There are significant differences in color and texture between the two. Since coal and gangue have a high degree of differentiation in appearance characteristics, it is scientific to extract the gray and texture features of coal and gangue images as indicators for distinguishing coal and gangue. Using formula (2) to calculate the probability of each gray level, and then draw the gray distribution histogram. In the formula, i is gray level ; n_i_ is the number of pixels with gray level i ; n is the total number of pixels ; hist (i) is the probability of i gray level occurrence.

2 $$Hist = \frac{{n_{i} }}{N}\left( {i = 0,1,2, \ldots ,255} \right)$$

**Figure 3:**
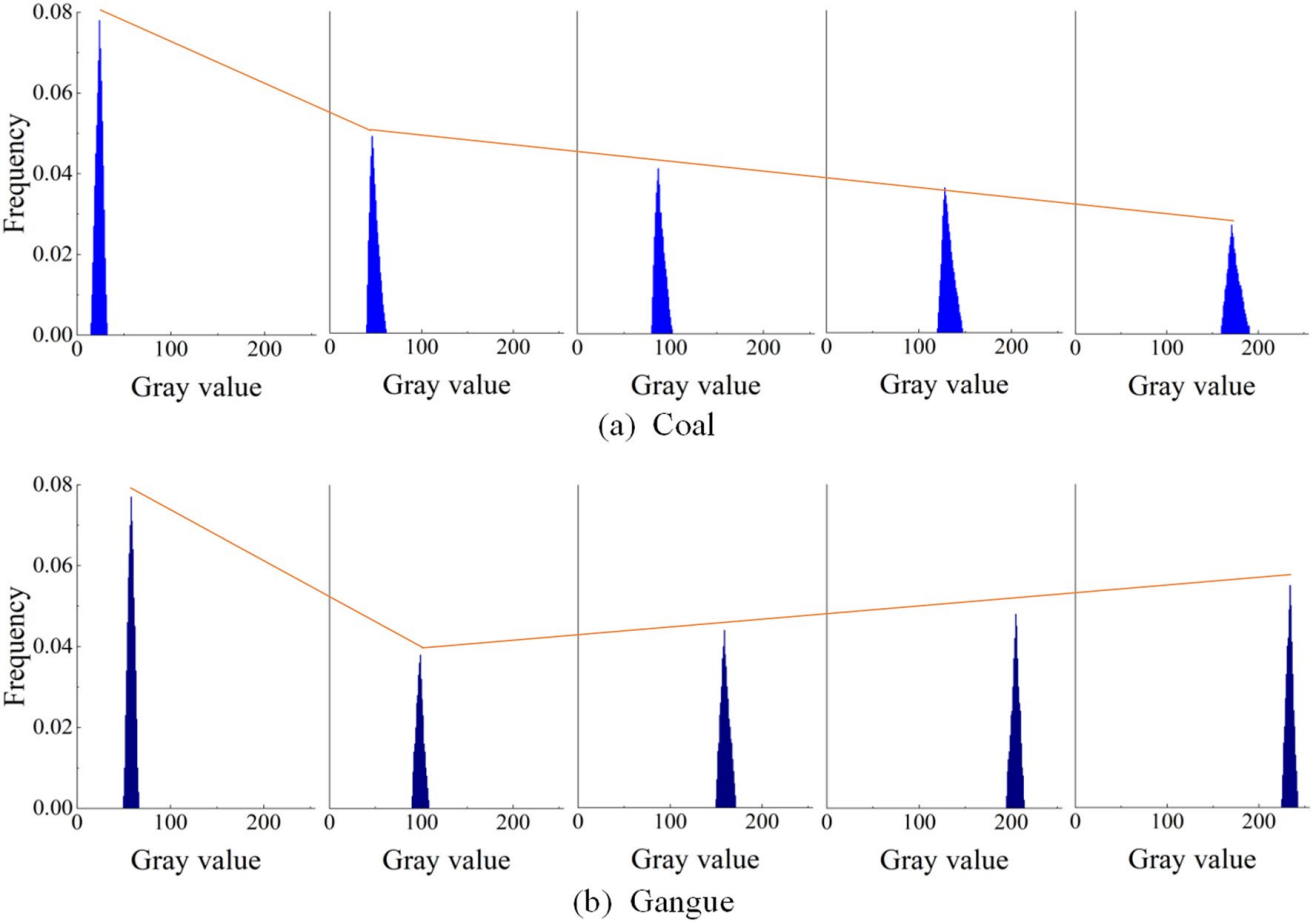
Gray distribution histogram of coal and gangue images.

When drawing the histogram of gray distribution of coal and gangue image, the smaller part of each group is deleted according to the statistical data of gray value. The purpose of this work is to make the gray distribution histogram more stereoscopic, which is conducive to observing the change trend of gray value distribution with the increase of illuminance, and to comparing the difference of gray value distribution under different illuminance conditions. As shown in Fig. 3.

It is found from Fig. 3a that the gray value of coal increases with the increase of illuminance in the experiment, which gradually increases from the initial 20–40 to 155–200. But the peak value is gradually reduced, and the image gradually becomes more “short and fat”, indicating that the gray value of coal is gradually dispersed, When the gray value of coal increases as a whole. The observation Fig. 3b found that with the increase of illumination in the experiment, the gray value of gangue is also increasing, but the increase is greater than that of coal, and the peak change shows a trend of first decreasing and then increasing. In addition, the gray distribution of gangue is more concentrated than coal, which is more “thin and high”. It can be concluded that the gray values of coal and gangue images increase with the increase of illuminance value, but with the increase of illuminance value, the change trend of the relationship between gray and frequency is inconsistent.

More importantly, by observing the gray distribution histogram, it can be seen that even under the same illumination, the gray values of coal and gangue also have obvious numerical differences, and with the change of illumination, the numerical differences of gray values between the two also change. Careful comparison found that under the condition of 17,130 Lux illumination, the gray value distribution of coal and gangue is farthest apart in the histogram. This shows that under this illumination condition, the gray value difference between coal and gangue is the largest, so it is concluded that 17130Lux is the best illumination condition for the feature extraction experiment of coal and gangue image.

## Grayscale image filtering and feature extraction of coal and gangue

### Gaussian filtering for grayscale images of coal and gangue

The most obvious distinction between coal and gangue images is the difference in gray and texture. In order to extract the gray and texture features of coal gangue images more accurately, it is necessary to optimize the gray images of coal and gangue obtained. In the process of image optimization, it is particularly important to remove the noise information in the image, because the noise information will “submerged” the original information characteristics in the image. In the process of image recognition, important information is often submerged by noise information. Noise information will interfere with the extraction and calculation of image feature parameters by computer, and affect the extraction of gray and texture feature values in coal and gangue gray images in this experiment. Therefore, removing noise information in coal and gangue gray image is the premise of extracting gray and texture feature values.

It is found that the noise in the gray images of coal and gangue mostly obeys the normal distribution, and the most effective way to eliminate this kind of noise is to conduct Gaussian filtering on the image. Although Gaussian filtering will have a certain impact on image quality, and it will make the image blurred to a certain extent, in essence, Gaussian filtering will hardly change the original effective information in the image, that is, it will not affect the extraction of relevant parameters of the image by the computer. Therefore, it is feasible and superior to conduct Gaussian filtering on the gray image of coal and gangue.

The principle of Gaussian filtering is to take a square window with new pixels as the center, and calculate the weighted average value of the original pixel value in the window. The weight of the original pixel value is determined by the distance between it and the center of the window, and the smaller the distance from the center, the greater the weight. Since the coal and gangue images in the experiment are two-dimensional images, two-dimensional Gaussian distribution function is needed to filter them. Formula (3) is the two-dimensional Gaussian distribution function

3 $$G\left( {{\text{x}},y} \right) = \frac{1}{{2\pi \sigma^{2} }}{\text{exp}}\left( { - \frac{{\left( {x - \mu_{x} } \right)^{2} + \left( {{\text{y}} - \mu_{y} } \right)^{2} }}{{2\sigma_{x} \sigma_{y} }}} \right) = G\left( x \right)G\left( y \right)$$

**Figure 4:**
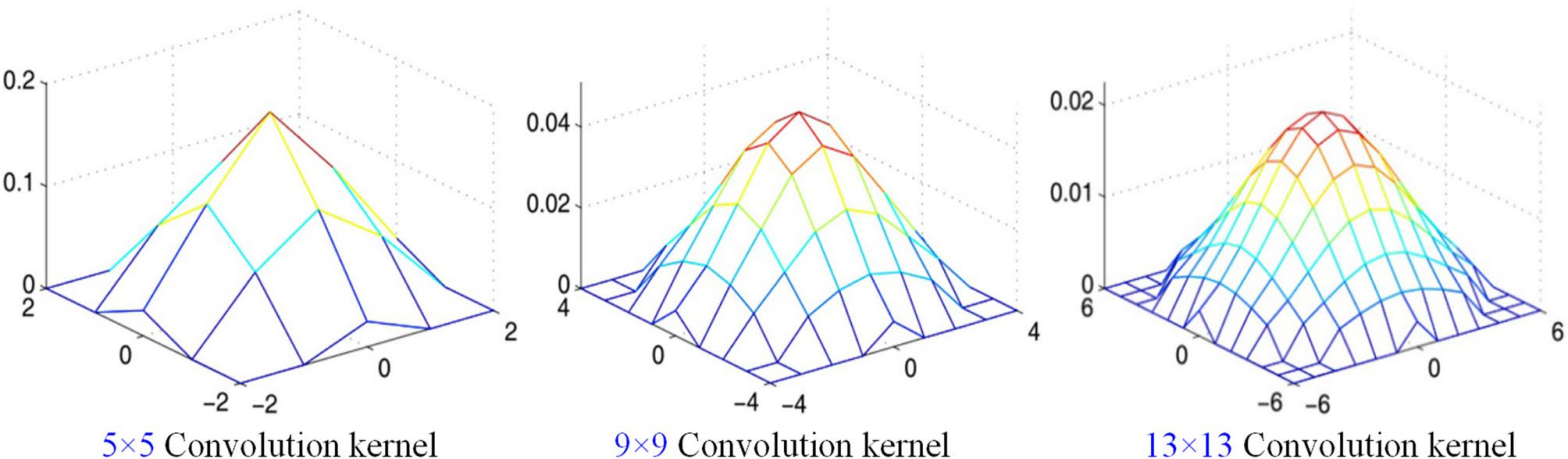
Two-dimensional Gaussian distribution function.

**Figure 5:**
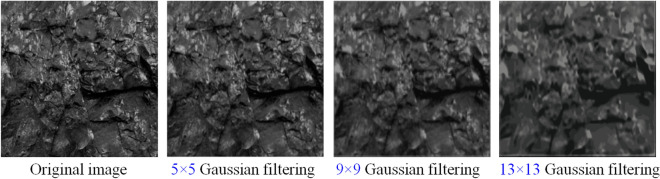
Processing results of coal ash image by Gaussian filter.

The Gaussian blur process of the image is the convolution between the image and the convolution kernel which obeys the two-dimensional normal distribution. In this paper, three different Gaussian kernel sizes of 5 × 5, 9 × 9, 13 × 13 are used. The corresponding two-dimensional Gaussian distribution function and the filtering effect of the image are shown in Fig. 4. The gray-scale sub-images of coal samples are selected for Gaussian filtering processing. Three different Gaussian kernel sizes of 5 × 5, 9 × 9 and 13 × 13 are used to observe the processing effect of gray-scale sub-images of coal samples, as shown in Fig. 5.

Observation Fig. 5 shows that there are obvious isolated pixels or pixel blocks (white particles) in the original gray image of coal samples. These noises in the high frequency part of the image affect the extraction of image related parameters on the one hand, and affect the processing speed of the computer on the other hand.Through the Gaussian filtering of the gray image of coal, it is observed that the isolated pixels in the image are gradually erased, that is, the large or small extremum in the digital image signal is erased. Convolution between the image and the circular convolution kernel in Gaussian filtering will generate more accurate out-of-focus imaging effect. As the noise is removed, the loss of image details does not decrease significantly.

With the removal of noise information, the loss of image details has changed to some extent. In the experiment, the gray image of coal is filtered by using three different Gaussian kernel sizes of 5 × 5, 9 × 9 and 13 × 13. The experimental results show that 9 × 9 Gaussian filter is the most suitable for processing the image of coal.

### Gray and texture features of coal and gangue images

Through the above experiments, the gray image of coal and gangue processed by Gaussian filtering is obtained. It is observed that coal and gangue have the most obvious difference in color and texture, coal is darker and gangue is more gray, which indicates that the grayscale characteristics of the two are significantly different. At the same time, the surface of coal has vitreous luster, and there are many cross-sections on the surface, which are prone to light reflection to form light spots, while the surface of gangue is more flat, with full or almost no reflection of light, and the texture characteristics of coal and gangue on this surface are also different.

There are many types of grayscale features and texture features, and the importance of various features in the field of coal gangue identification is also different. It is related to the extraction of image features that can be adjusted at any time according to the quality of the extraction results. parameters or to further optimize the extraction method. In this experiment, the method of manually extracting image features is more conducive to obtaining the most effective experimental results. In order to realize the recognition of coal and gangue by image recognition algorithm, the specific digital quantity is usually used to describe the difference of gray and texture between coal and gangue images. The experiment selected the following 10 kinds of statistics as quantitative description of coal and gangue image gray, texture features.

The gray mean represents the average gray value of the image. Visually, the larger the gray mean is, the greater the image brightness is. The formula is as follows. In the formula, G-1 represents all the gray values in the image, z_k_ represents the gray level, n_k_ represents the number of times each gray level appears, N is the total number of pixels.

4 $${\text{Mean}} = \sum\limits_{K = 0}^{G - 1} {{\text{z}}_{{\text{k}}} } \frac{{n_{k} }}{N}$$

Gray variance represents the dispersion degree of gray value. Visually, the larger the gray variance is, the greater the image contrast is, that is, the greater the color difference between pixels is.The formula is as follows : p(z_k_) represents the probability of gray z_k_ appearing in the image, M represents the mean gray value.

5 $$Variance = \sum\limits_{k = 0}^{G - 1} {\left( {z_{k} - M} \right)}^{2} p\left( {z_{k} } \right)$$

Gray skewness represents the degree of asymmetry of image gray distribution, and its formula is as follows. In the formula, is the gray variance, i is the gray level, and n_i_ is the number of pixels in the gray level i.

6 $$Skewness = \frac{1}{{\sigma^{3} }}\sum\limits_{i = 0}^{255} {\left( {i - \mu } \right)}^{3} \frac{{n_{i} }}{N}$$

The gray peak indicates the degree to which the gray distribution is concentrated near the gray mean, and the formula is as follows.

7 $$Peak = \frac{1}{{\sigma^{4} }}\sum\limits_{i = 0}^{255} {\left( {i - \mu } \right)}^{4} \frac{{n_{i} }}{N} - 3$$

Gray energy represents the uniformity of image distribution, and its formula is as follows.

8 $$Energy = \sum\limits_{i = 0}^{255} {\left( {\frac{{n_{i} }}{N}} \right)}^{2}$$

Gray entropy represents the uneven and chaotic degree of gray distribution, and its formula is as follows.

9 $$E{\text{ntropy}} = - \sum\limits_{i = 0}^{255} {\frac{{n_{i} }}{N}} \times \log_{2} \frac{{n_{i} }}{N}$$

**Figure 6:**
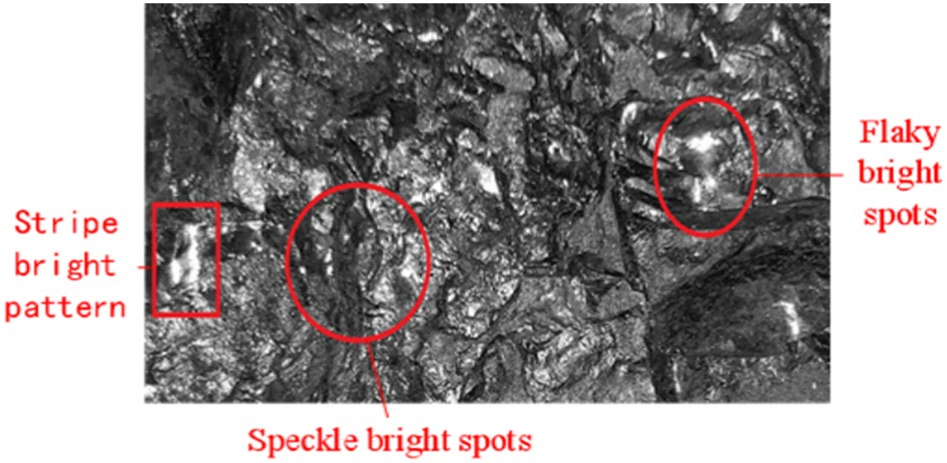
Bright spots formed by reflection on coal surface.

By observing coal and gangue, it is found that coal is more metallic luster than gangue, and its reflection is better. Moreover, bright spots or bright lines will appear on the surface or cross section of coal when the reflection occurs. Determined by the physical structure of gangue, the light absorption of gangue is better than that of coal, and its reflection is naturally worse than that of coal. Some gangues with special texture have almost no reflection. Therefore, it is also feasible to study texture features to realize coal gangue identification, and the reflectance of coal is shown in Fig. 6.

Texture is a visual feature that reflects the homogeneity phenomenon in the image, which reflects the arrangement property of surface structure with slow or periodic changes on the surface of the object. Texture feature is a regional feature. Different from gray feature, it is not for a single pixel to be analyzed, but for a number of pixels in a certain region of the image to be uniformly calculated.

In this paper, four independent texture features are studied based on gray level co-occurrence matrix (GLCM) .Texture contrast describes the gray level difference between adjacent pixels in the image, the formula is as follows. The matrix P (i, j, l, α) represents all α directions of the pixel (x, y), i and j represent the values of two adjacent pixels, *l* represents the spacing of adjacent pixels, and the direction α is usually 0°, 45°, 90° and 135°.

10 $$Conrtast = \sum\limits_{n = 0}^{255} {n^{2} } \left\{ {\sum\limits_{i = 0}^{255} {\sum\limits_{j = 0}^{255} {p\left( {i,j,l,\alpha } \right)^{2} } } } \right\}$$

Texture correlation describes the similarity of gray levels between adjacent pixels in the image, and the formula is as follows, α represents the gray variance, u_1_, u_2_ represents the gray mean of two adjacent pixels.

11 $$Correlation = \sum\limits_{i = 0}^{255} {\sum\limits_{j = 0}^{255} {\frac{{ijP\left( {i.j,l,\alpha } \right) - u_{1} u_{2} }}{{\sigma_{1}^{2} \sigma_{2}^{2} }}} }$$

Texture Angle Second Moment (ASM) describes the uniformity of pixel gray level distribution, and the formula is as follows.

12 $$ASM = \sum\limits_{i = 0}^{255} {\sum\limits_{j = 0}^{255} {p\left( {i,j,l,\alpha } \right)} }^{2}$$

Texture homogeneity Similarly describes the uniformity of pixel gray level distribution, the formula is as follows.

13 $$Homogenetiy = \sum\limits_{i = o}^{255} {\sum\limits_{j = 0}^{255} {p\left( {i,j,l,\alpha } \right)} } /\left[ {1 + \left( {i - j} \right)^{2} } \right].$$

## Experiment on extracting gray and texture eigenvalues of coal and gangue images

A total of 1800 images of coal and gangue were obtained by graying and Gaussian filtering. The specific differences of gray and texture features between coal and gangue images are studied experimentally. Finally, “effective features” are selected as indicators to distinguish coal and gangue. According to the above six kinds of gray features and four kinds of texture features, the relevant eigenvalues of coal and gangue images are extracted.

**Figure 7:**
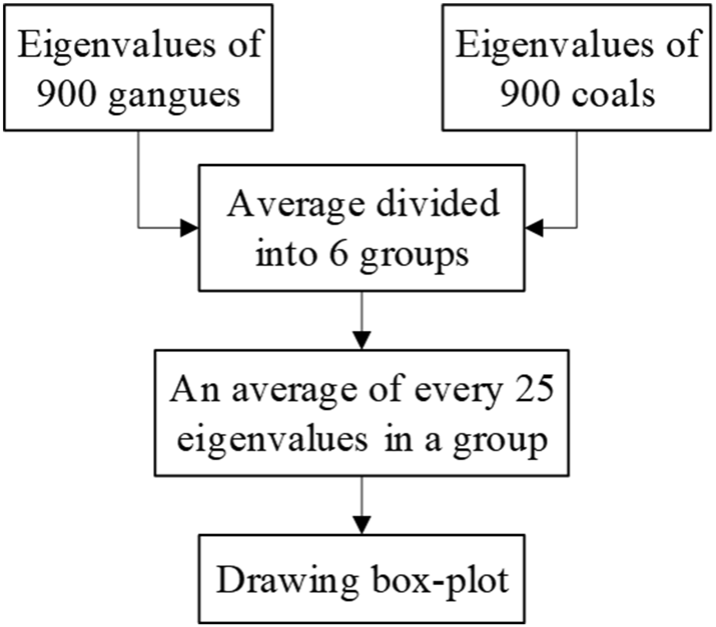
Eigenvalue processing flow.

In order to ensure the universality and persuasiveness of the experimental results, the experimental samples need to be divided into six groups for feature extraction at the same time. In the experiment, 900 coal images and 900 gangue images were divided into six groups with the same number, and labeled as one group, two groups, three groups, four groups, five groups and six groups. Ten eigenvalues were extracted for each coal or gangue image, and a total of 18,000 eigenvalues were obtained. According to the different types of eigenvalues, the eigenvalues are classified and studied. For a type of eigenvalues, according to the group label, 150 sub-data in the group are divided into 6 data sets on average, and the arithmetic average of 25 eigenvalues in each data set is calculated. For a type of gray or texture features, the feature values of six groups of samples are extracted at the same time and the statistical diagram is drawn to ensure that the experimental results are universal and persuasive. The experimental process of eigenvalue data is shown in Fig. 7.

In the experiment, it is found that the individual data of some experimental groups are significantly larger or smaller than those of other data in the same group. Considering the particularity of the study on the difference of coal and gangue characteristics, the rationality of its existence cannot be ruled out without clarifying the causes of abnormal values. In order to objectively and completely reflect the experimental results, each group of data is drawn into a box-plot for display.

**Figure 8:**
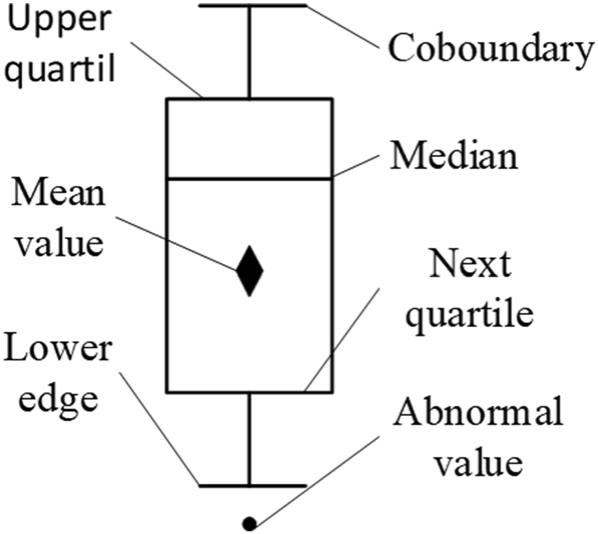
Principle of box-plot.

When the number of statistical data is large and highly dispersed, it is more reasonable to choose the box-plot as the data statistics. box-plot is used to display a set of statistical maps of data dispersion. It reflects the characteristics of the original data distribution, and can also compare the distribution characteristics of multiple sets of data. The principle of box drawing is shown in Fig. 8.

### Grayscale features

The differences between coal and gangue in various gray and texture features were studied experimentally. The feature values of a large number of coal and gangue images were extracted to select the features with large numerical differences as “effective features” and use such features as indicators to distinguish coal and gangue. In order to analyze the characteristic value data in the box-plot and study the discrimination of various characteristics on coal and gangue images, the difference rate is introduced to study it. The formula is as follows. In the formula, A represents the eigenvalues of coal, B represents the eigenvalues of gangue, D_R_ represents the difference rate between the two. The larger the D_R_ value is, the greater the degree of difference between the images of coal and gangue is for such eigenvalues, which also confirms that such eigenvalues have high discrimination between coal and gangue.

14 $$D_{R} = \frac{{\left( {A - B} \right)}}{B} \times 100\%$$

**Figure 9:**
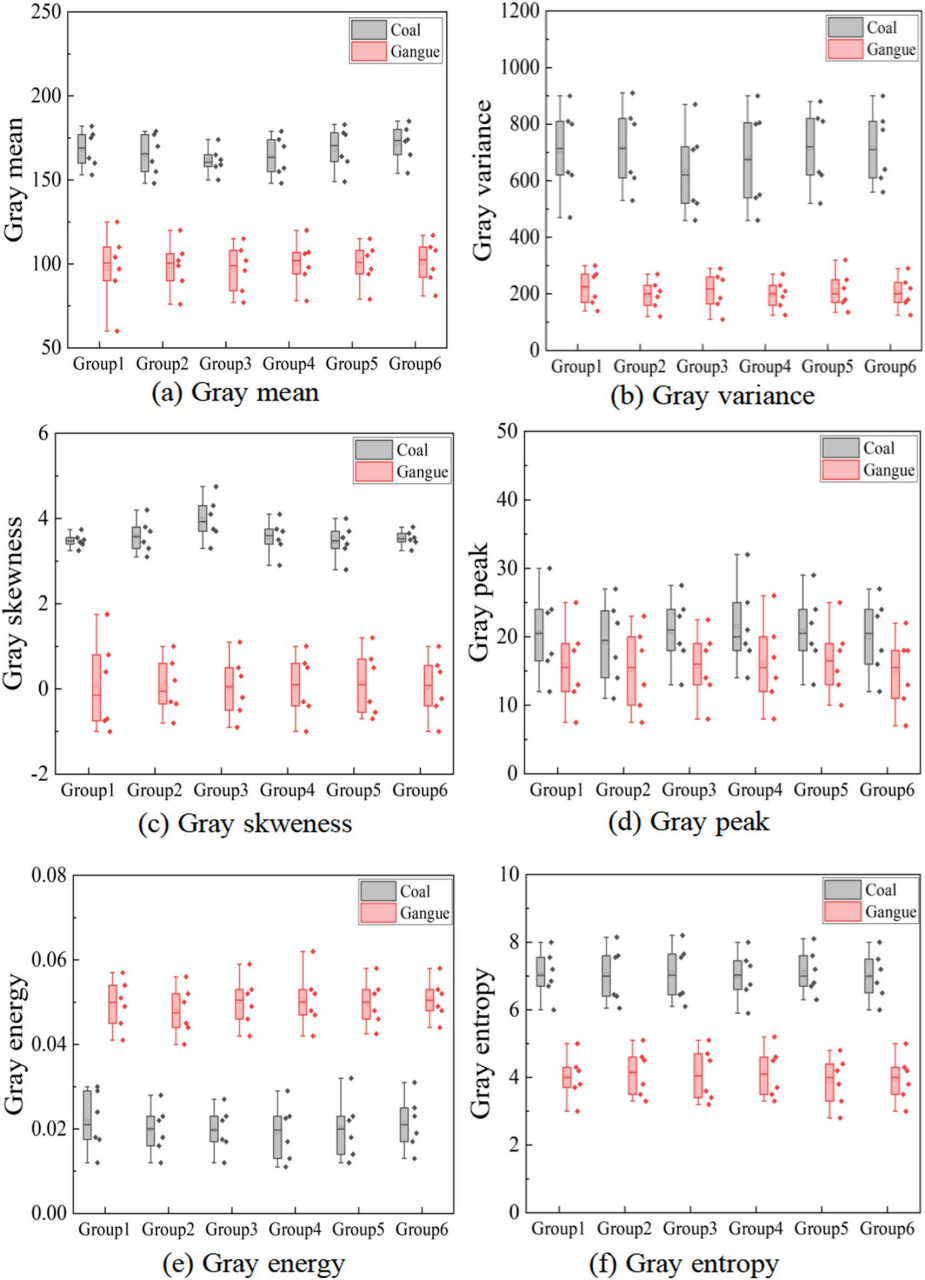
Gray characteristic values of coal and gangue.

In order to ensure the universality and persuasiveness of the experimental results, six groups of coal and gangue images with the same number were selected for feature extraction experiments, and the obtained data were plotted into a box-plot. The six gray feature extraction results of coal and gangue are shown in Fig. 9.

According to the, the box-plot distribution area and distribution characteristics of various gray values of coal and gangue images can be intuitively seen. By studying the distribution of eigenvalues, the discrimination of each type of gray value on coal and gangue images can be analyzed. The eigenvalue with the farther difference in numerical distribution proves that the difference between coal and gangue images in the nature of this kind of gray feature is larger.

The observation Fig. 9 shows that there are some differences in the six gray characteristic values of coal and gangue images. The box-plot analysis shows that the gray mean, gray variance and gray skewness are the highest for the discrimination of coal and gangue, and the gray peak state is the lowest for the discrimination of coal and gangue, because the distribution of its eigenvalues is consistent and the difference is not large. In order to accurately select the effective features, the statistical data in the box-plot is calculated by formula (14). The experimental results show that the difference rate of gray mean value between coal and gangue is 70%, the difference rate of gray variance is 250%, the difference rate of gray peak state is 33%, the difference rate of gray skewness is 600%, the difference rate of gray energy is 150%, and the difference rate of gray entropy is 56%.

From the calculation results, it can be concluded that among the gray features of coal and gangue, the difference rates of gray contrast, gray variance and gray skewness are the highest, which proves that these three gray features have the highest discrimination against coal and gangue, and are most suitable for the identification of coal and gangue, belonging to effective features. The gray peak state has the lowest discrimination between coal and gangue, which is classified as invalid feature. At the same time, the experimental data of six sample groups are statistically studied to ensure the universality of the experimental results.

### Texture feature

**Figure 10:**
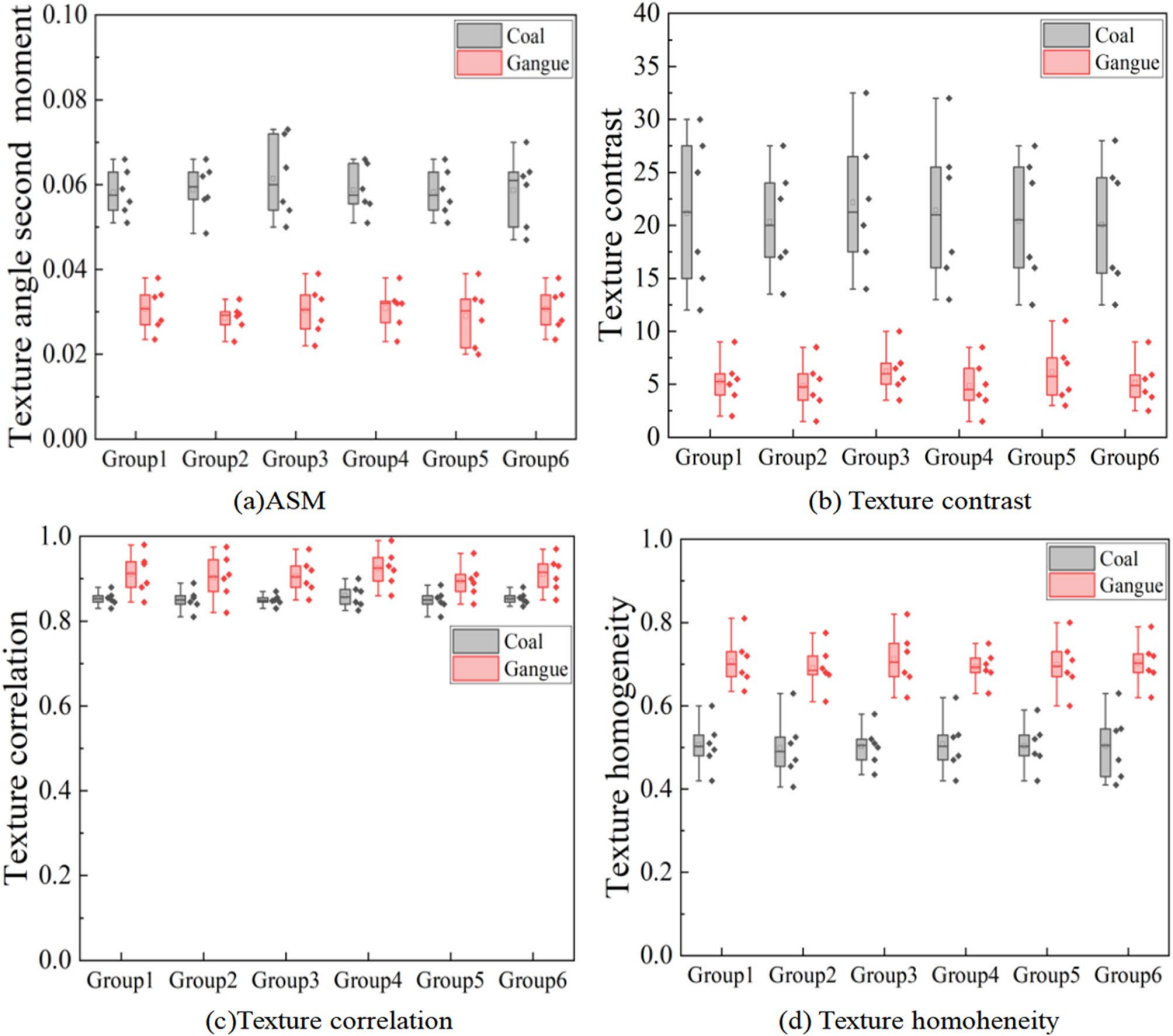
Texture eigenvalues of coal and gangue.

In order to study whether the four texture features of coal and gangue images have differences in distribution and the degree of difference, the experiment uses the same way to extract the four texture eigenvalues of coal and gangue images respectively, and the eigenvalue data are drawn into a box-plot The distribution of four texture eigenvalues of coal and gangue is shown in Fig. 10. It is found in Fig. 10 that there are some differences in the distribution of four texture eigenvalues of coal and gangue images. Intuitively, texture contrast and texture angle second-order moment have the highest discrimination between coal and gangue, while texture correlation has the lowest discrimination between coal and gangue. In order to accurately select effective features, the statistical data in the box-plot are calculated by formula (14) . The results show that the difference rate of texture contrast between coal and gangue is 300%, the difference rate of texture angle second moment is 100%, the difference rate of texture homogeneity is 40%, and the difference rate of texture correlation is 5.6%.

From the calculation results, the texture contrast and the second-order moment difference rate of texture angle of coal and gangue are the highest, which proves that the two texture features have the highest discrimination on coal and gangue, and are the most suitable discriminant basis for coal and gangue identification, belonging to effective features. The texture correlation is invalid features, because the experiment carried out data statistics on six sample groups at the same time, which can confirm the universality of the experimental results.

## Coal and gangue image recognition model

### Characteristic difference Indexefwr

In order to analyze the effect of various gray and texture features as indicators for coal gangue identification more clearly and intuitively, the same features of coal and gangue are grouped as one feature. According to Formula (15), the eigenvalues of each group are normalized to [0, 1]. In the formula, Y represents the eigenvalues of different categories, and V_Yi_ is the eigenvalues of group i in the Y feature group (i = 1–6) ; V_Ymax_ and V_Ymin_ are the maximum and minimum eigenvalues in each group. f_Yi_ (v) is the output result of eigenvalues (0 or 1).

15 $$f_{Yi} \left( V \right) = \frac{{V_{Yi} - V_{Y\min } }}{{V_{Y\max } - V_{Y\min } }}$$

The normalized characteristic difference index (NFDI) is defined, as shown in Eq. (16), which indicates the relative difference between the features of coal and gangue. The larger the value is, the higher the difference between coal and gangue is. On the contrary, the smaller the value is, the lower the difference between coal and gangue is. In the formula, NFDI_Yi_ is the normalized coal gangue characteristic difference index of the i sample group.

16 $$NFDI_{{Y{\text{i}}}} = \left| {f_{Yic} \left( V \right) - f_{Yig} \left( V \right)} \right|$$

**Figure 11:**
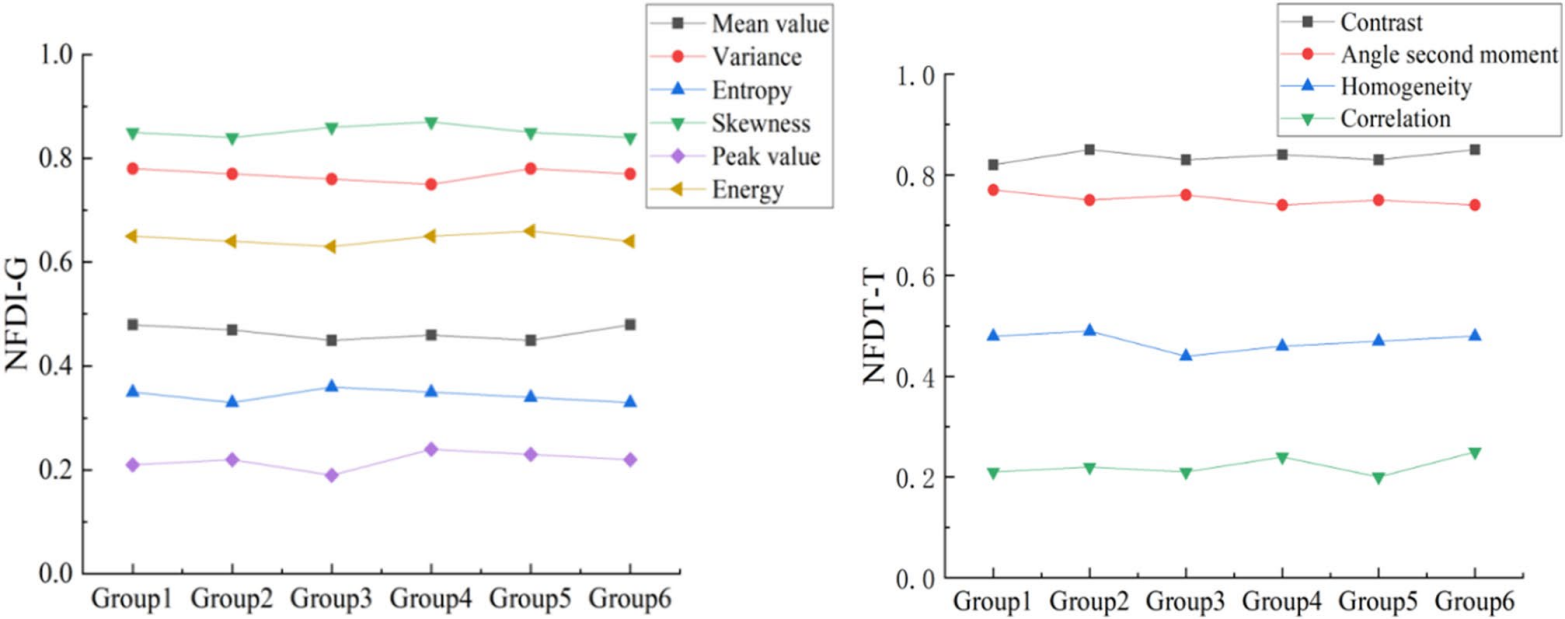
Difference index of coal and gangue characteristics in different groups.

In order to facilitate the comparison of eigenvalue data, NFDI-G is named as gray feature difference, and NFDI-T is called as texture feature difference. Differences in gray and texture features among different groups are shown in Fig. 11.

### Mechanism of LS-SVM model

Through the analysis of the images of coal and gangue samples, it is found that although there are differences between the gray features and texture features of coal and gangue samples, the coal and gangue are mixed together in the actual coal and gangue sorting process, resulting in the linear inseparable gray features and texture features of some coal and gangue. Therefore, the identification of coal and gangue by nonlinear classifier can obtain better recognition rate. LS-SVM converts the inequality constraints of SVM into equality constraints, which greatly reduces the difficulty of solving hyperplanes and the time consumption in the solving process, and effectively improves the solving efficiency of the algorithm. Support vector machine as a typical two-class classifier, there are mainly one-to-one and one-to-many two models. The actual research object of this experiment is the identification of coal and gangue, and the selected classifier type is one-to-one. The LS-SVM model is used to identify coal gangue images according to various gray and texture features.

In order to improve the recognition rate of coal gangue in LS-SVM model, the original space of LS-SVM is as follows : ω represents high dimensional feature space vector, b represents classification threshold, ζ represents relaxation factor, C represents punishment factor, *l* = 1,2,3… n.

17 $$\min J\left( {\omega ,b,\xi } \right) = \frac{1}{2}\left\| \omega \right\|^{2} + \frac{1}{2}C\sum\limits_{l = 1}^{n} {\xi_{l}^{2} }$$

In order to optimize the model, the Lagrange function is used, in which α represents the Lagrange multiplier.

18 $$L\left( {\omega ,b,\xi ,\alpha } \right) = J\left( {\omega ,b,\xi } \right) - \sum\limits_{l - 1}^{n} {\alpha_{l} } \left\{ {y_{l} \left[ {\omega^{T} \varphi \left( {x_{l} } \right) + b} \right] + \xi_{l} - 1} \right\}$$

The partial derivatives of ω, b, ζ_*l*_, α are obtained respectively, and then the linear equations can be obtained according to the KKT optimization condition. The decision function can be obtained by solving the linear equations in the original space as follows.

19 $$y\left( x \right) = {\text{sgn}} \left[ {\sum\limits_{l - 1}^{n} {\alpha_{l} y_{l} K\left( {x,x_{l} } \right) + b} } \right]$$

Symmetric functions satisfying Mercer ' s theorem can be used as the kernel function of SVM. Radial basis function (RBF) can classify samples very close, and only contains a broad factor σ. In this paper, exponential RBF is used as the kernel function.

20 $$K\left( {x,x_{l} } \right) = \exp \left( { - \frac{{\left\| {x - x_{l} } \right\|^{2} }}{{\sigma^{2} }}} \right)$$

The penalty factor C affects the classification accuracy of SVM, while the broad factor σ of RBF affects the learning ability and classification performance of SVM. The optimal value is selected by fivefold cross validation. The Libsvm toolbox of MATLAB is used for simulation, and it is found that the recognition rate of coal gangue is the highest when C = 11 and σ = 1.

**Figure 12:**
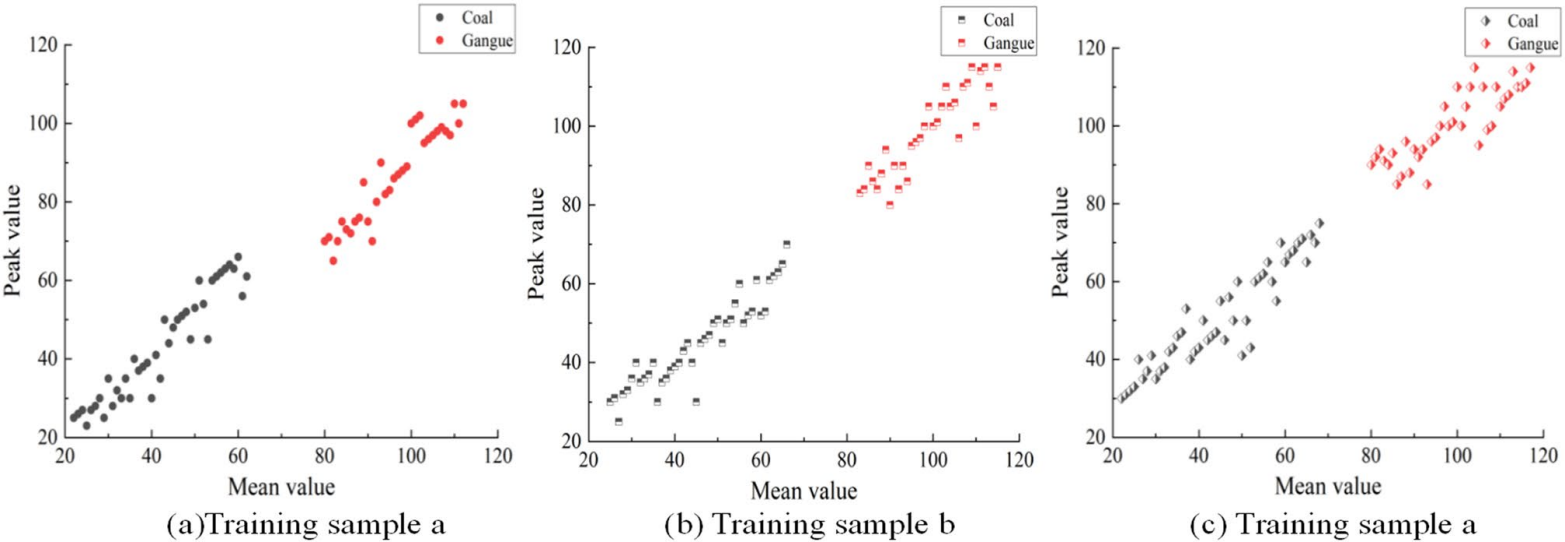
Gray feature classification view of coal and gangue.

The above coal and gangue images were divided into three groups on average to train the model, with labels of a, b and c, respectively. Each group contains 600 images of coal gangue. When training the model, the labels of coal and gangue are set to 0 and 1 respectively. Next, the gray feature data of coal and gangue are normalized. Finally, the LS-SVM model is trained by using the svmtrain ( ) function. LS-SVM model is used to identify coal and gangue samples in three sample sets. Figure 12 shows the classification view of LS-SVM model trained by the gray features of coal and gangue samples in three sample sets. The black image in Fig. 12 represents the LS-SVM model to identify the image as coal, and the red image represents the image as gangue. The horizontal axis is the average gray value, and the vertical axis is the peak gray value. The classification hyperplane trained by LS-SVM model with gray features of three coal and gangue samples can separate coal and gangue samples to the maximum extent. It is necessary to construct a machine model for coal gangue identification by using gray and texture features. When the model inputs the images of coal and gangue, the output result is [0, 1], where 0 indicates that the model is identified as coal, and 1 indicates that the model is identified as gangue. LS-SVM classifier is called least squares support vector machine. This algorithm enables the model to learn effectively in the case of a small number of samples, and can complete the effective prediction of unknown samples with high accuracy.

**Figure 13:**
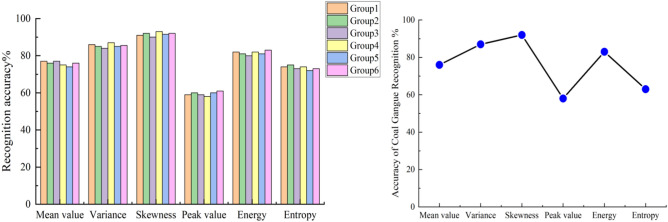
LS-SVM classifier recognition results based on gray feature.

**Figure 14:**
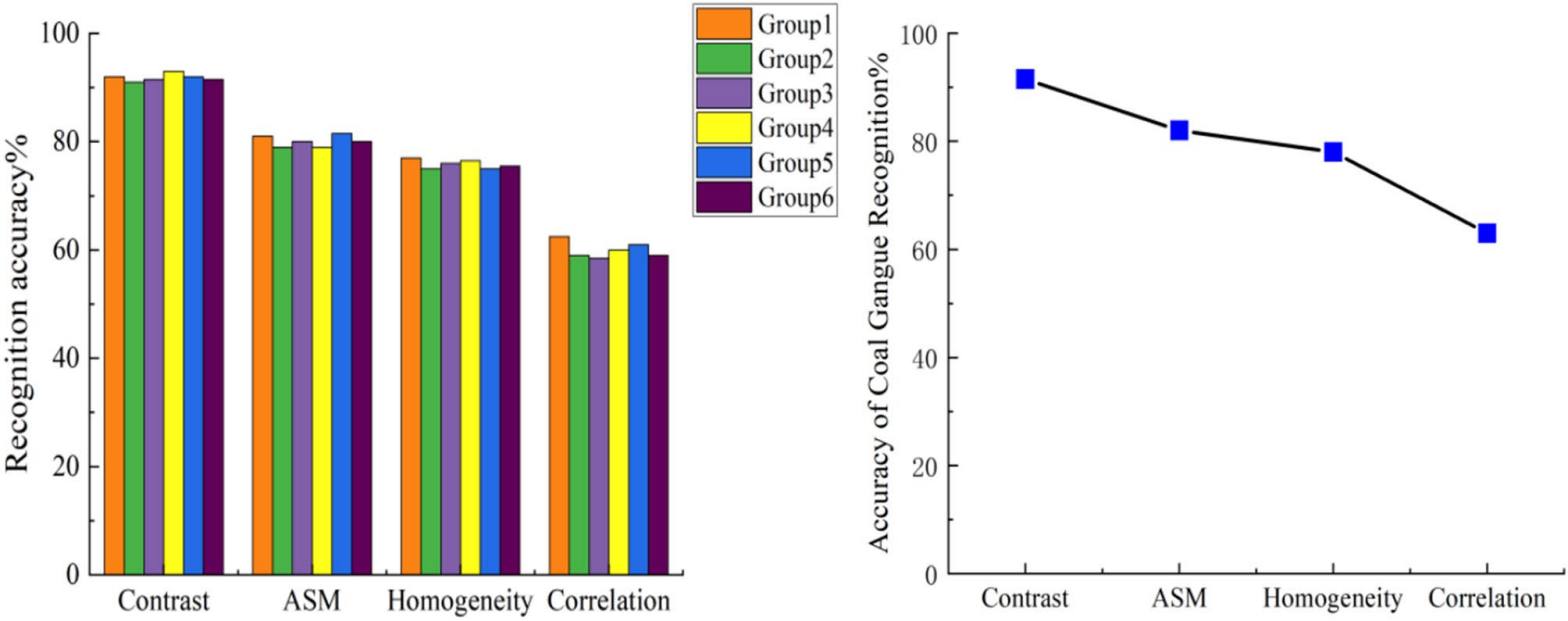
LS-SVM classifier recognition results based on texture features.

A total of 1800 images in the coal and gangue image database were identified according to different feature types by using the LS-SVM classifier, and the recognition accuracy is shown in Figs. 13 and 14. Due to the different properties of each gray feature and texture feature, the recognition accuracy of LS-SVM model is also different.

Through the illumination experiment of coal and gangue, 17,130Lux is determined as the optimal illumination condition for extracting gray and texture features from coal and gangue images. Therefore, the coal and gangue samples should be photographed under this illumination to construct the image database of coal and gangue. Since image processing is mostly independent of its color attributes, in order to speed up its processing speed, the coal and gangue images are grayed. The analysis shows that there are many noise information obeying the normal distribution in the gray image of coal and gangue, so Gaussian filter with the best noise suppression effect is introduced to denoise the image. According to the experimental results, 9 × 9 Gaussian filter is selected to filter the gray images of coal and gangue.

In the experiment of extracting gray and texture feature values, six groups of experiments were conducted for each type of feature at the same time, which ensured the universality of the experimental results.The difference degrees of six gray features and four texture features of coal and gangue images were experimentally studied. By calculating the difference rate, the gray energy, gray contrast, gray variance and texture skew were selected as the effective features of coal and gangue identification.

**Figure 15:**
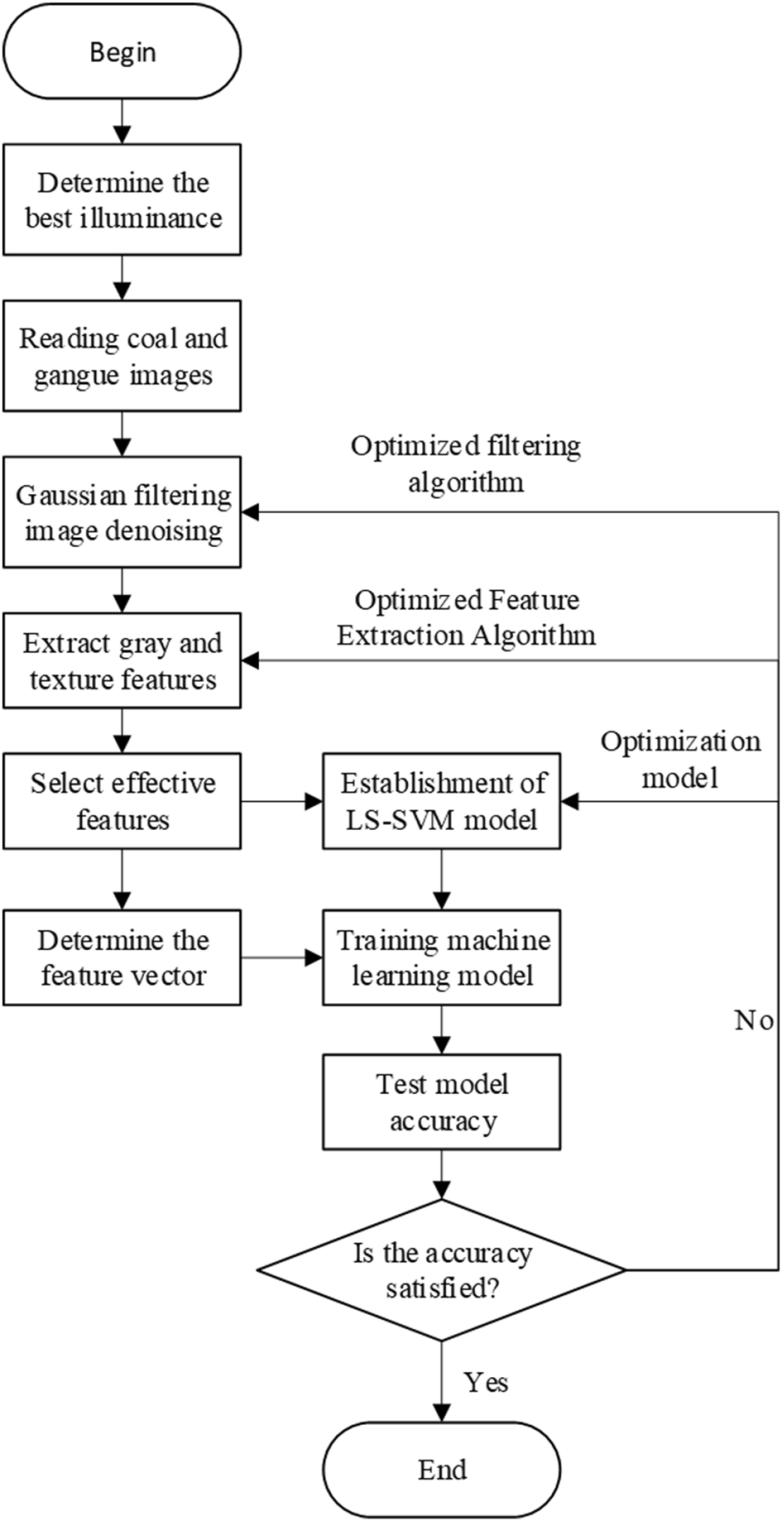
Flow chart of coal gangue identification system.

The LS-SVM classifier is used to construct the coal gangue recognition model. The output is 0 or 1, 0 indicating that the model recognition image is coal, and 1 indicating that the model recognition image is gangue. At the same time, a certain number of training sets are input to improve the accuracy of coal gangue recognition. Through the above research, a coal gangue recognition system based on gray and texture features can be constructed, and the flow chart is shown in Fig. 15. Firstly, the system collects the coal and gangue images under the illumination value of 17,130 Lux and grays them. Then, the acquired images are processed by 9 × 9 Gaussian filtering to eliminate the noise information in the image. Then, the gray features and texture features in the image are extracted and the effective features are selected. The LS-SVM model is constructed and trained while determining the feature vector, and the recognition accuracy of the model is finally tested. If the accuracy does not meet the expected requirements, the Gaussian filtering algorithm or the feature extraction algorithm or the optimization model are optimized until the recognition accuracy of the model reaches the expected requirements.

## Conclusions

1. Illumination is an important factor affecting image quality and related parameters. The multi-light source illumination system is designed to collect coal and gangue images under different illumination conditions, and then the gray distribution histogram is drawn. By observing and analyzing the histogram of gray distribution, it is concluded that under the illumination of 17130Lux, the gray difference between coal and gangue is the highest, so 17130Lux is determined as the best illumination condition for shooting coal and gangue images.
2. Experiments on coal and gangue image gray processing, without losing image gradient information while improving the computer processing speed. The analysis shows that there are many noises in the coal and gangue images that obey the two-dimensional normal distribution. After studying the effect of several Gaussian filtering, 9 × 9 Gaussian filtering is selected to filter coal and gangue images, which is beneficial to the extraction of eigenvalues of coal and gangue images.
3. Through the extraction of six kinds gray features and four kinds texture features, a total of 18,000 feature values were drawn into a box-line diagram for study in six groups. Through the analysis, it is concluded that the three characteristics of gray skewness, gray variance and texture contrast have the highest discrimination between coal and gangue. These three parameters will be important indexes to realize coal gangue identification.
4. The LS-SVM model was constructed in the experiment, and the collected 1800 coal and gangue images were used to form a training set for training to improve the recognition accuracy of the model for coal gangue images. The trained model was used to identify 18,000 eigenvalues in the experiment, and finally the recognition accuracy was counted and the statistical diagram was drawn. It is concluded that the model has the highest recognition accuracy of 92.2% for gray skewness and the lowest recognition accuracy of 61.7% for gray variance. In terms of texture features, the model has the highest recognition accuracy for texture contrast, which is 91.5%, and the lowest recognition accuracy for texture correlation, which is 57.1%.

## Data Availability

The data used to support the findings of this research are included within the paper.
